# Effect of preheat repetition on color stability of methacrylate- and silorane-based composite resins

**DOI:** 10.15171/joddd.2017.039

**Published:** 2017-12-13

**Authors:** Mehdi Abed Kahnamouei, Sarah Gholizadeh, Sahand Rikhtegaran, Mehdi Daneshpooy, Soodabeh Kimyai, Parnian Alizadeh Oskoee, Yashar Rezaei

**Affiliations:** ^1^Dental and Periodontal Research Center, Tabriz University of Medical Sciences, Tabriz, Iran; ^2^Department of Restorative Dentistry, Tabriz University of Medical Sciences, Tabriz, Iran; ^3^Department of Restorative Dentistry, Ahvaz Jundishapur University of Medical Sciences, Ahvaz, Iran

**Keywords:** Composite resin, preheating, microleakage, color stability

## Abstract

***Background.*** The aim of this study was to investigate the effect of preheating methacrylate- and silorane-based composite resins on their color stability up to 40 times at 55‒60°C.

***Methods.*** Seventy-six methacrylate and silorane-based composite resin samples, with a diameter of 10 mm and a height of 2 mm, were divided into 4 groups (n=19). After the samples were prepared, their color parameters were determined using a reflective spectrophotometer. The composite resin samples were separately stored in a solution of tea for 40 consecutive days. Then the samples underwent a color determination procedure again using a spectrophotometer and color changes were recorded. Finally two-way ANOVA was used to study the effect of composite temperature on its staining (P<0.05). Independent-samples t-test was used to evaluate changes in conversion rates of preheated composite resin samples compared to non-heated samples at P=0.005 and P=0.029 for silorane-based and Z250 composite resin samples, respectively.

***Results.*** Both composite resin type (P=0.014) and preheating (P<0.001) had significant effects on ΔE.

***Conclusion.*** Repeated preheating of methacrylate- and silorane-based composite resin samples, up to 55‒60°C for 40 rounds, resulted in more color changes compared with unheated composite resin samples. After storage in a solution of tea the color change rate in the composite resin samples of silorane-based was higher than the Z250 composite resin samples.

## Introduction

Improvements in modern composite resin properties have led to the use of these materials in various dental restorative procedures.^1^ Since the introduction of composite resins, many efforts have been made to increase their efficiency in the oral cavity as a restorative material; however, the optical specifications of composite resins still require further studies.^2,3^

The change in composite resin color is a multifactorial phenomenon of intrinsic or extrinsic origin. intrinsic factors include alterations in the chemical composition of the resin matrix and the interface between the filler particles and the matrix, while extrinsic factors consist of absorption of colored materials from external sources connected to patient’s oral hygiene habits, food and smoking cigarettes.^4^ It should be pointed out that the propensity of the resin matrix for discoloration is under the influence of conversion rate and the physicochemical properties of the matrix and the amount of water absorbed.^5,6^ Many clinical and laboratory investigations have shown a relation between the degree of conversion and composite resin properties.^7^

The degree of monomer conversion affects the chemical stability of the substance. Non-converted dual carbon bands are not only capable of making the material disposed to bond destruction, resulting in a decrease in color stability and release of materials such as methacrylic acid and formaldehyde.^8-10^ It also facilitates the influence of solvents from the oral environment on the polymer network, destroying recently formed chains.^8^

Recent studies have focused on heating composite resin to improve its properties.^11‒14^ Heating composite resin before photopolymerization not only decreases its viscosity but also has the advantage of improving its mechanical properties, including an increase in conversion rate and composite resin surface hardness.^15,16^ In addition, preheating increases the flow of composite resin, increasing adaptation of composite resin with cavity walls, which finally results in a decrease in microleakage and extrinsic staining of the restoration.^11,16^ During preheating, the composite syringe is heated prior to use at a temperature range of 39‒60°C.^17^ However, many studies have shown that preheating does not have any destructive effects on the mechanical properties of composite resins.^18-20^ In the studies cited, mechanical specifications have been evaluated for only one thermal cycle, while in a clinical setting, a composite syringe is often used several times to restore multiple cavities, which will result in various preheating cycles if preheating technique is used.^21^

Recent studies have focused on heating composite resin to improve its properties.^11,14^ Heating composite resin before photopolymerization not only decreases its viscosity but also has the advantage of improving its mechanical properties, including an increase in conversion rate and composite resin surface hardness.^15,16^ In addition, preheating increases the flow of composite resin, increasing adaptation of composite resin with cavity walls, which finally results in a decrease in microleakage and extrinsic staining of the restoration.^11,16^ During preheating, the composite syringe is heated prior to use at a temperature range of 39‒60°C.^17^ However, many studies have shown that preheating does not have any destructive effects on the mechanical properties of composite resins.^18-20^ In the studies cited, mechanical specifications have been evaluated for only one thermal cycle, while in a clinical setting, a composite syringe is often used several times to restore multiple cavities, which will result in various preheating cycles if preheating technique is used.^21^

The relationship between degree of conversion and temperature increase in several composite resin systems has been investigated.^9,12,13^ However, few studies have been carried out on the effect of preheating on optical properties of composite resins.^2,3^ A study by Mundin et al on the effect of preheating on degree of conversion of Tetric N Ceram nano-hybrid composite resin showed that despite an increase in the conversion rate, preheating had no influence on the optical properties of composite resin.^22^

Considering what was discussed above and the importance of chemical differences of resin components in the color stability of composite resin,^23,24^ the aim of this study was to investigate the effect of 40 cycles of preheating on the color stability of silorane- and polymethacrylate-based composite resins.

## Methods

Filtek P90 and Filtek Z250 composite resins with A3 shade were used in the present study. The brands, manufacturers and chemical compositions of composite resins used in the present study are presented in Table 1. Seventy-six methacrylate- and silorane-based composite resin samples, with a diameter of 10 mm and height of 2 mm, were divided into 4 groups (n=19): group 1: 19 methacrylate-based composite resin samples at room temperature; group 2: 19 methacrylate-based composite resin samples preheated 40 times^17^ up to 55‒60°C; group 3: 19 silorane-based composite resin samples preheated 40 times^17^ up to 55‒60°C; group 4: silorane-based composite resin samples preheated 40 times^17^ up to 55‒60°C. Each preheating cycle included the time required to heat composite resin up to a temperature of 55‒60°C and cool down to room temperature (which lasted 12 minutes).^17^ Disk-shaped samples of composite resin were prepared for each group using plastic molds with a diameter of 10 mm and a height of 2 mm uniformly and standardized. The material was placed on a slab of glass and then, using a composite resin plastic instrument, small pieces of composite material were placed within the mold. Two blocks of glass were placed under and over each mold during preparation of the samples to prevent the formation of non-polymerized composite resin layer and to achieve a smooth surface layer. After placing a sufficient amount of composite rein in each mold, another glass block was placed on it. Then, the samples were cured from each side for 40 seconds each at a light intensity of 700 mW /cm^2^ with a light-curing unit (Astralis 7, Ivocolar Vivadent FL-9494 Schaan, Liechtenstein). The light intensity of the equipment was checked periodically with a radiometer (Demetron LED Radiometers, Kerr Restoratives, Italy). The surface layer of the composite resin samples, which was to be evaluated by a spectrophotometer in relation to its color, was distinguished by placing a mark by a diamond fissure bur on the contralateral surface. Then excess light-cured composite resin was removed from the samples. In the next stage, silicon carbide paper disks (Soflex-3M ESPE, Ultrathin, USA) were used up to 1000 grit to polish the sample surfaces to achieve a uniform surface and eliminate all the possible contaminations. It was expected that polishing would simulate the clinical conditions of composite restorations, so that the color changes reported at the end of the study would be attributed only to the inherent properties of composite resin.^ (25)^ Calipers were used to make sure of the thickness homogeneity in all the samples and all its areas. The final thickness of the samples was 2 mm after polymerization, finishing and removal of the samples from the molds.

**Table 1:** The brands, manufacturers and chemical compositions of composites used in research

**Material**	**Composition**	**Manufactured by**
**Filtek™ Z250 universal composite**	Filtek™ Z250 universal restorative : Matrix composition: resin consisting of BIS-GMA (Bisphenol A diglycidyl ether dimethacrylate), Bis-EMA (Bisphenol A polyethylene glycol dietherdimethacrylate) and UDMA (urethane dimethacrylate). Filler particle: 60% (volume) silica/zirconia	3M ESPE Dental Product U.S.A
**Filtek** ^TM^ **Silorane, low shrink composite**	Filtek^TM^ silorane –based composite: Matrix composition: siloxane and oxirane (23% of the composition). Inorganic filler: fine quartz particles, yttrium fluoride (76% ) .	3M ESPE Dental Product U.S.A

After these steps, all the prepared samples were stored in distilled water for 48 hours at 37°C to bring about initial water sorption, complete polymerization and simulate the oral cavity conditions.^25^ After preparation of the samples and transferring them into distilled water, a reflexive spectrophotometer (Spectraflash 600, Data Color International, USA) was used to determine color parameters of the samples and recording the parameters used in the CIE system: *L (luminescence), *a (red/green) and *b (blue/yellow) as the baseline parameters. In order to prepare a solution of tea, a Lipton tea bag (Lipton is a universal brand for tea, Made in England) was immersed in 150 mL of boiling water and left to cool to room temperature. The composite specimens were kept 3 hours per day in the tea solution for 40 consecutive days. The solution was replaced and refreshed every day.^25^

Then the samples were subjected to the spectrophotometry technique once again to determine the color of the samples and the color changes were recorded. The procedural steps in groups 2 and 4 were similar to those in groups 1 and 3, with the difference that prior to the use of composite resin, it was initially heated to temperatures of 55‒60°C by floating in a thermostatically controlled heated water bath, which was set at a temperature of 55‒60°C.^26^ Then the composite resin was injected into the mold, followed by steps similar to those in group 1.

In order to evaluate the conversion rate, before placement of the samples in tea solution, 4 samples were taken from each group randomly and evaluated, using Fourier Transform Ramon Spectrometer (ALMEGEGA Dispersive Raman, Thermo Nicolet, USA). Two-way ANOVA (P<0.05) was used to study the effect of composite resin temperature on degree of staining. For evaluating changes in conversion rates of preheated composite resin samples compared to non-heated samples, independent-samples t-test was used at P=0.005 and P=0.029 for silorane-based and Z250 composite resin samples, respectively.

## Results

The results showed that the color changes in silorane-based composite resin were significantly greater than those of Z250, with significantly more color changes in preheated composite resin compared to un-heated composite resin (resins used in the present study are presented in Tables 2 and resins used in the present study are presented in 3). Two-way ANOVA declared that both the main effects of heat (P<0.001) and composite (P=0.014) on ΔE were significant.

**Table 2:** Means, standard deviations, and maximum and minimum values in both composite resin types before and after heating

		**No.**	**Mean**	**SD**	**Min**	**Max**
**Silorane**	Heated	19	14.9653	1.61799	12.03	17.81
	Unheated	19	12.7253	2.19959	8.51	19.36
**Z250**	Heated	19	13.3353	2.41234	10.14	18.96
	Unheated	19	11.9847	1.89317	8.77	17.49

**Table 3:** Two-way ANOVA to study the main effects of type of composite resin and heat and their cumulative effect on color changes (ΔE)

**Source**	**Type III sum of squares**	**P-value**
**Composite resin**	26.692	.014
**Heat**	61.236	.000
Composite resin*heat	3.758	.348

**Figure 1:**
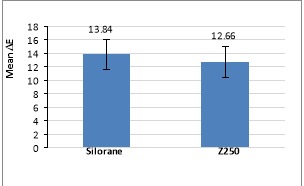
Comparison of ΔE between the two composite resin types.

Comparison of ΔE between silorane-based composite resin and Z250 composite resin with two-way ANOVA revealed significantly more color changes in silorane-based composite resin (P<0.05). In other words, there was significant staining in heated silorane-based composite resin after immersion in tea solution (Figure 1).

**Figure 2:**
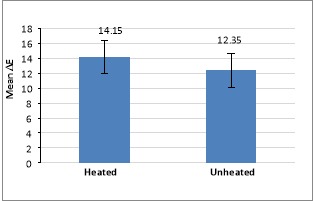
Comparison of ΔE before and after heating.

Comparison of mean ΔE before and after heating with two-way ANOVA showed that the color change in the preheated composite resin was significantly higher than that in unheated composite resin (P<0.05) (Figure 2). In addition, comparison of color changes between silorane-based and Z250 composite resins before and after heating, at a constant of 3.3, revealed significant differences (P<0.05).

Investigation of the degree of conversion of preheated composite resin groups compared to unheated groups with independent-samples t-test showed a significant difference between them (Table 4).

**Table 4:** Comparison of conversion rates between preheated and unheated composite resin samples

**Composite**	**Temperature**	**Mean**	**Sig (2-tailed)**
**Silorane**	Heated 55‒60°C Unheated 25°C	1.49‏±0/048 1.29‏±0/048	0.02
**Z250**	Heated 55‒60°C Unheated 25°C	0.76‏±0/037 0.65‏±0/037	0.11

## Discussion

The results of this study showed that the color change in the two composite materials studied was significant in terms of the type of composite and applied heat.

Immersion of composite resin samples heated to 55‒60°C in tea solution for 40 days resulted in significantly more staining in heated the groups (2 and 4) compared to the un-heated corresponding groups (1 and 3), and comparison of preheated groups (2 and 4) revealed that staining in silorane-based composite resin samples was more severe than methacrylate-based composite resin samples.

To overcome the composite resin polymerization shrinkage problems, low-shrinkage composite resins have been introduced recently.^27^ Short-term studies have shown that these composite resins may exhibit favorable mechanical and optical properties; nevertheless, long-term clinical and laboratory investigations are needed to better understand the clinical behavior of these composite resins.^28^

A new study showed that all the physical and mechanical properties of these newly introduced composite resins are not satisfactory,^29^ as confirmed by the conclusion of the this study on the color stability of silorane-based composite resins.

Color changes of ΔE<1.5 cannot be distinguished by human eye; however, ΔE>3.3 is considered unacceptable clinically.^30,6^ The composite resins evaluated in the present study after immersion in a solution of tea exhibited color changes higher than the those acceptable clinically, with the maximum staining in P90 composite resin, which was significantly different from that of Z250 composite resin, contrary to the results of studies by Arocha^ (31)^ and Barutcigil,^32^ in which comparisons of methacrylate- and silorane-based composite resins were performed after dipping them in different colored solutions. Silorane-based composite resin showed less coloration than the methacrylate-based composite resin. Also, Palin's study results were attributed to less water sorption by silorane-based composite resin,^33^ which resulted in less severe staining. In Palin's study, the effect of heating composite resin before immersion in colored solutions was not studied. However, in the present study, the effect of staining of composite resin specimens after 40 preheat cycles was investigated and it appears the discrepancies between the results of the present study and those of the studies above might be attributed to preheating.

In the present study, staining of the silorane group was significantly higher than the methacrylate group, consistent with a study by Pires-de-Souza,^34^ in which staining of the silorane composite after artificial aging process was higher than methacrylate composite resins. One of the factors responsible for such a finding is separation of quartz particles from the resin matrix during the artificial aging process, leading to doubts about the effect of silane on the bond between the quartz particles and the epoxy silorane base to induce an effective and long-term bond. In Pires-de-Souza's study, micrography assessments demonstrated that separation of quartz particles from the resin matrix during the artificial aging process creates an irregular surface.^34^ The covalent bond among the glass particles and the resin matrix significantly influences the composite resin properties.^35^

In addition, in the present study, the severity of staining in both Z250 and P90 composite resin groups was significantly less than that before heating, which might be explained by the significant increase in conversion rate in both groups after 40 rounds of preheating, consistent with a study by Mundin.^22^ The curing temperature has a significant effect on the degree of composite resin conversion.^11^ Preheating of composite resins through enhancing radical mobility and a decrease in system viscosity influences polymerization and increases degree of conversion, affecting color stability.^10,36^ Heating composite resin before photopolymerization not only decreases its viscosity but also has the advantage of improving mechanical properties, including an increase in conversion rate and composite resin surface hardness.^15,16^ In addition, preheating increases the flow of composite resin, increasing adaptation of composite resin with cavity walls, which finally results in a decrease in microleakage and extrinsic staining of the restoration.^11,16^ The conversion rate of the monomer affects the chemical stability of the substance. Non-converted dual carbon bonds are capable of making the material disposed to bond destruction, reducing color stability and releasing materials such as methacrylic acid and formaldehyde.^8‒10^ It also facilitates the influence of solvents from the oral environment on the polymer network and destroys recently formed chains.^8^ In addition, color stability and stain susceptibility are directly influenced by polymerization values.^10,12^ In the current study, preheating composite resin samples for 40 cycles increased degree of conversion, but in spite of the increased degree of conversion, color changes in heated study groups (2 and 4) were more than that in the unheated study groups (1 and 3).

Under clinical conditions, two important factors involved in preheating of composite resins are composite resin temperature when placed in the cavity and the time interval between removal of the composite resin from the compoule and its placement in the cavity. Preheated composite resin might cool rapidly, negating the advantages of preheating.^22^ However, based on the results of a study by Nic et al,^37^ even if composite resin is cooled to 40°C, its advantages can still be helpful compared to composite resin cooled to room temperature.

Daronch et al^38^ reported that the intra-pulpal temperature in vitro increased after placement of preheated composite resin. In this context, an increase of 0.8°C was recorded through 1 mm of wet dentin after placement of composite resin preheated to 60°C. They also recorded an increase of 5°C during photopolymerization of composite resin and concluded that after removing the composite resin from the heater, the temperature dropped rapidly to 36°C. In other words, the results did not show significant differences in intra-pulpal temperatures with preheated composite resin and composite resin at room temperature.

Bagheri^30^ study reported that tea is one of the beverages that causes more discoloration than the others. In the current study, presence of the tea solution and the weakened covalent bonds between the quartz particles and the epoxy resin of silorane-based composite resin can cause more staining in silorane-based composite resin. In conclusion, these color changes are considered unacceptable clinically (ΔE>3.3).^6,30^

A study by Amario^17^ on the effect of 20 and 40 rounds of preheating on the flexural strength of composite resins showed that 20 cycles of preheating did not result in significant differences from the unheated controls; however, after 40 cycles the differences were significant. In the present study, color changes were evaluated after 40 cycles of preheating and significant differences were detected but it was not clear whether the differences between 20 and 40 cycles would be significant. It can be concluded from the results of this study that repeated cycles of preheating have a negative effect on the color stability of composite resins. If clinicians are aware of the fact that they would use a composite syringe for more than 40 times with the preheating process, it would be preferable to use disposable composite resin instead of syringes based on the results of the current study.

## Conclusion

Repeated preheating of methacrylate- and silorane-based composite resin samples to 55‒60°C for 40 rounds resulted in more color changes compared with unheated composite resin samples. After storage in a solution of tea color changes in silorane-based composite resin specimens were much higher than those in the Z250 composite resin specimens.

## Acknowledgements

The authors express their grateful thanks to the head of department for providing general support and Dr. M. Ghojazadeh for statistical analysis.

## Authors’ contributions

MAK was responsible for the study design and supervised the study; SG was the owner of the thesis, prepared the samples, wrote the article writing and analyzed data; SR and MD helped in the preparation of samples; SK assisted in writing the article writing and in its publication; YR assisted in revising the English text and collecting the samples; PAO gave advice in article publication and provided scientific advice. All authors critically revised the manuscript and have read and approved the final version.

## Funding

This study was financially supported by Tabriz University Medical Sciences.

## Competing interests

The authors declare no competing interests with regards to the authorship and/or publication of this article.

## Ethics approval

Not applicable.
